# Imaging in Neuro-oncology

**DOI:** 10.1055/a-2719-5058

**Published:** 2025-10-30

**Authors:** Elizabeth Coffee, Cleopatra Elshiekh, Joshua A. Budhu

**Affiliations:** 1 Department of Neurology, Memorial Sloan Kettering Cancer Center, New York, New York, United States; 2 Department of Neurology, Weill Cornell Medicine, New York, New York, United States; 3 Immigrant and Cancer Disparities Service, Department of Psychiatry, Memorial Sloan Kettering Cancer Center, New York, New York, United States

**Keywords:** neuro-oncology, brain tumor, advanced MRI, amino acid PET, radiomics

## Abstract

Brain tumors are a diverse group of neoplasms that vary widely in treatment and prognosis. Imaging serves as the cornerstone of diagnosis, monitoring response to treatment and identifying progression of disease in neuro-oncologic care. This review outlines current and emerging imaging modalities with a focus on clinical application in glioma, meningioma, and brain metastasis. We cover standard imaging modalities, advanced magnetic resonance techniques such as perfusion and spectroscopic imaging, and nuclear imaging with positron emission tomography (PET), including amino acid PET. We summarize the standardized Response Assessment in Neuro-Oncology (RANO) criteria, and explore innovations in radiomics, artificial intelligence, and targeted imaging biomarkers. Finally, we address challenges related to equitable access to advanced imaging. This review provides a practical, clinically focused guide to support neurologists in the imaging-based care of patients with primary or metastatic brain tumors.

## Introduction

Central nervous system (CNS) tumors represent a diverse group of primary and secondary neoplasms that vary widely in clinical behavior, prognosis, and treatment strategies. Primary brain and spine tumors arise from cells within the CNS and include gliomas (astrocytomas, oligodendrogliomas, glioblastomas), meningiomas, pituitary adenomas, primary central nervous system lymphomas (PCNSL), and rarer histologies such as medulloblastomas and ependymomas. Glioblastoma, which originates from glial cells, is the most common primary malignant brain tumor in adults, with over 13,000 new cases diagnosed per year. 1 Meningiomas are the most common primary brain tumors, with approximately 40,000 diagnosed per year. 1

Secondary brain tumors, or brain metastases, are far more common than primary CNS tumors and occur in up to 20 to 40% of patients with systemic cancer, particularly lung, breast, melanoma, renal cell carcinoma, and colorectal cancer. 2 Improved CNS surveillance and imaging, along with more efficacious systemic therapies, are leading to a growing number of patients with brain metastases. 3

Neuroimaging is one of the most frequently used tools in neuro-oncology and often serves as the first step in diagnosing or identifying CNS tumors. Clinicians use imaging not only to confirm the presence of a tumor but also to guide surgical and radiation planning. Throughout treatment and follow-up, imaging helps monitor tumor response, detect recurrence, and identify treatment-related effects. After therapy, neurologists rely on imaging to assess disease progression and make informed adjustments to the care plan.

However, interpreting imaging findings in neuro-oncology is often complex. Treatment-related phenomena such as pseudoprogression, transient increase in lesion enhancement due to inflammation after chemoradiation, and pseudoresponse, rapid reduction in enhancement and edema following anti-angiogenic therapy, can mimic true disease progression or response. These effects complicate radiographic assessments and highlight the importance of clinical context and the integration of advanced imaging techniques. In response to these challenges, neuroimaging has advanced significantly, with new modalities offering deeper insight into tumor biology, treatment response, and prognosis. 4 The growing complexity of imaging technologies and interpretation frameworks underscores the need for a practical understanding of how to apply these tools in neurologic care.

This review outlines both current and emerging imaging modalities in neuro-oncology and demonstrates how neurologists can apply these tools in clinical practice. It covers core techniques, advanced magnetic resonance imaging (MRI) sequences, and nuclear imaging, including positron emission tomography (PET). We summarize standardized response criteria such as the Response Assessment in Neuro-Oncology (RANO) and explore innovations in radiomics, artificial intelligence, and targeted imaging biomarkers. Finally, we address challenges related to equitable access to advanced imaging, an increasingly important issue as precision medicine continues to evolve. This review provides a practical, clinically focused guide to support neurologists in the imaging-based care of patients with primary or metastatic brain tumors.

## Core Imaging Modalities

### Computed Tomography (CT)

Computed tomography (CT) serves as a rapid, accessible imaging modality, often used in the acute setting when MRI is not always feasible or practical. Clinicians commonly utilize CT at initial presentation, in response to acute changes in the clinical picture and postoperative surveillance. At initial presentation, CT is used to rule out ischemic stroke, hemorrhage, and CNS infection. Features of intra-axial tumors are widely variable. Many demonstrate hypoattenuation or isoattenuation, with CNS lymphoma and meningiomas being the most common hyperattenuated masses. Tumors tend to spare the cortex and demonstrate mass effect and edema, which may look more severe than presenting symptoms would suggest. Clinicians rely on CT to triage patients with midline shift, ventricular entrapment, and concern for increased intracranial pressure. Bony involvement is rare but can be seen in some invasive tumor types. Unexplained calcifications may also raise suspicion for primary brain tumors. CT is also used for operative planning, often with fiducial markers to support image-guided neuronavigation, and intraoperative CT can provide real-time updates to guide resection and confirm surgical accuracy. 5 6 In any case where tumor is suspected, clinicians should pursue MRI of the brain with and without contrast.

### Magnetic Resonance

#### Core Sequences

Magnetic resonance represents the mainstay of radiologic assessment for both primary and secondary brain tumors. Although clinical symptoms play an important role, radiologists and clinicians rely on MRI to assess both progression of disease and treatment response. A consensus for Brain Tumor Imaging Protocol (BTIP) was developed in 2015 7 and while geared toward clinical trial standardization, it has been widely adopted at most brain tumor centers. This includes axial T2-weighted sequences, axial fluid-attenuated inversion recovery (FLAIR), axial diffusion-weighted imaging (DWI), and 3D T1-weighted pre- and post-contrast images using either a 1.5T or 3T strength magnet.

**Fig. 1:**
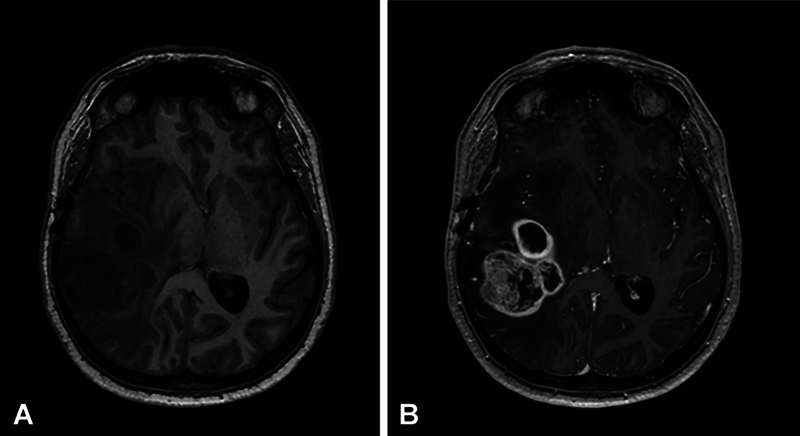
Axial T1-weighted pre-contrast image ( **A** ) and post-contrast image ( **B** ) of a glioblastoma. The pre-contrast image shows a mix of hypointense T1 signal in the right hemisphere. In post-contrast image, there is a heterogeneously enhancing tumor with irregular signal characteristics, consistent with necrosis, angiogenesis, and intratumoral blood products.

Contrast-enhanced MRI remains the most sensitive and reproducible imaging modality to assess brain tumors, establishing it as the standard imaging approach 8 ( Fig. 1 ). Due to the characteristic angiogenesis associated with more aggressive tumors, pre- and post-contrasted T1-weighted images are critical in any brain tumor evaluation. 3D isotropic imaging allows the detection of smaller lesions as well as more accurate comparison between scans to detect changes in lesion size by limiting differences in slice prescriptions. An added advantage is the ability to reconstruct images in different planes which are often needed for neurosurgical or radiation planning, without the need for reacquisition. Although not yet standardized, multiple studies 9 10 11 have demonstrated the benefit of using volumetric assessments over conventional cross-sectional areas for tumor assessment, particularly in nonenhancing tumors. 12 Volumetrics can decrease inter-use variability in size assessments and help detect early signals of progression when conventional radiographic response criteria are not yet met. 13

**Fig. 2:**
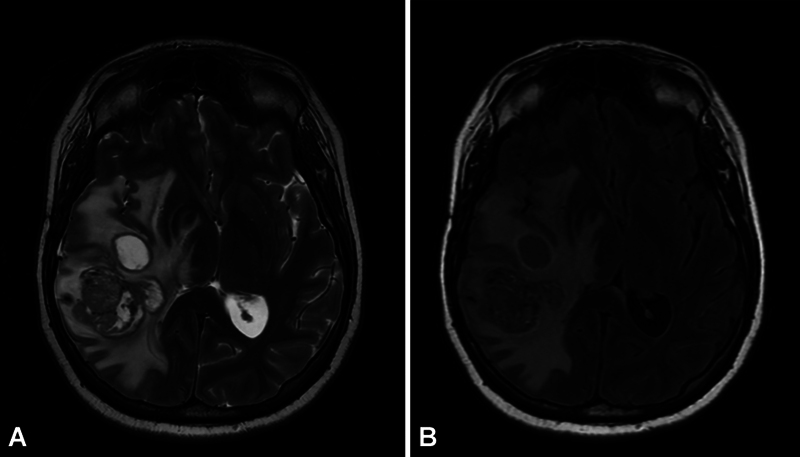
Axial T2-weighted image ( **A** ) and T2-weighted fluid-attenuated inversion recovery (FLAIR) image ( **B** ) of the same glioblastoma shown in Fig. 1. Note the peritumoral hyperintense T2 signal, indicative of edema. On the T2-weighted image, cerebrospinal fluid (CSF) appears hyperintense, with signal intensity similar to the necrotic, cystic regions within the tumor. In contrast, the FLAIR sequence suppresses the CSF signal, enhancing visualization of the tumor relative to normal brain parenchyma.

T2-weighted images are crucial for monitoring nonenhancing tumors such as low-grade gliomas, as well as early indicators of progression in enhancing tumors and those treated with anti-angiogenic agents such as bevacizumab. 14 T2-weighted FLAIR MRI combines T1- and T2-weighting to suppress CSF signal, allowing improved visualization of periventricular and juxtacortical tumor, vasogenic edema, and gliosis ( Fig. 2 ). FLAIR sequences are used to determine progression in the Response Assessment in Neuro-Oncology (RANO) criteria, which is detailed in a later section.

**Fig. 3:**
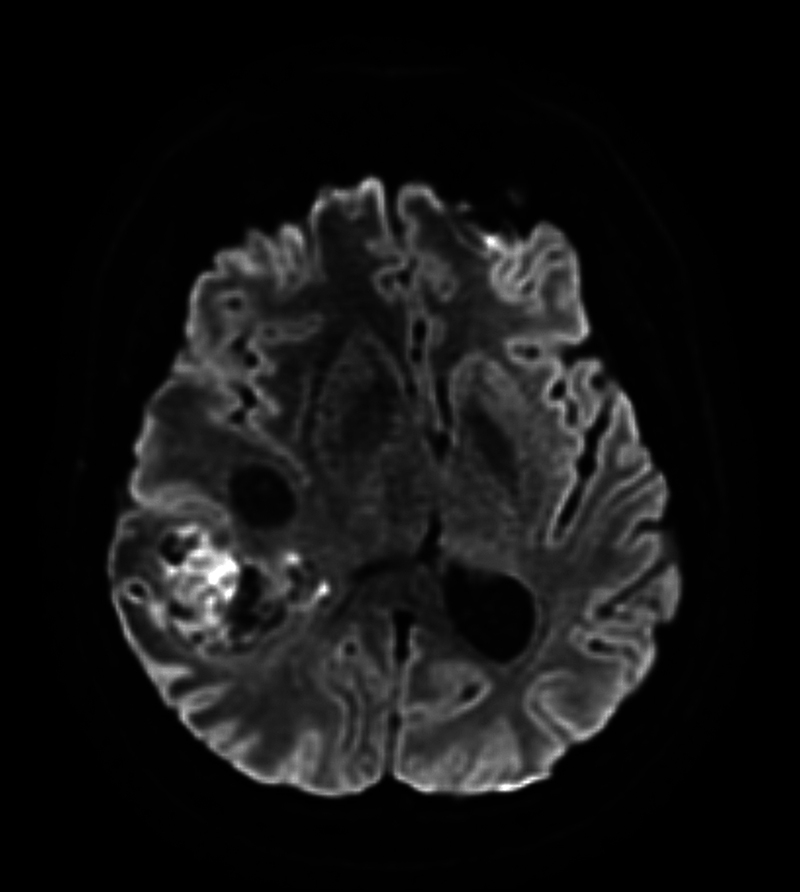
Axial T2-weighted diffusion-weighted imaging (DWI) of the glioblastoma shown in Figs. 1 and 2. The hyperintense signal within the tumor indicates areas of hypercellularity and restricted water diffusion.

DWI is the last component of standard tumor imaging protocols ( Fig. 3 ). Its sensitivity to microscopic water motion allows visualization of infection as well as ischemic injury. Because DWI is acquired with T2-weighting, high T2 signal can appear bright on DWI (“T2 shine through”); thus, an apparent diffusion coefficient (ADC) map is often generated by comparing DWI of two distinct *b* -values. ADC values have been inversely correlated with cellular density, which aids in distinguishing between grades of glioma and highly cellular tumors such as lymphoma from primary gliomas. 15 DWI and ADC have been investigated in predicting overall survival 16 and distinguishing tumor progression from treatment effect 17 18 19 ; however, the presence of vasogenic edema and necrosis causes substantial variation in these measurements, limiting their widespread use.

## Advanced MRI

### Perfusion

Three primary methods for measuring hemodynamic perfusion have been studied in neuro-oncology: dynamic susceptibility contrast MRI (DSC-MRI), dynamic contrast-enhanced MRI (DCE-MRI), and arterial spin labeling (ASL). DSC measures the drop in T2 signal intensity over time as a function of local contrast concentration over time, generating dynamic measurements such as relative cerebral blood volume (rCBV). DSC is the most commonly used perfusion imaging and is widely incorporated into research and clinical studies. It is particularly useful in distinguishing CNS lymphoma from primary glioma, where the former demonstrates lower rCBV, 20 and solitary brain metastases from infiltrative glioma where the peritumoral region often shows increased rCBV. 21

**Fig. 4:**
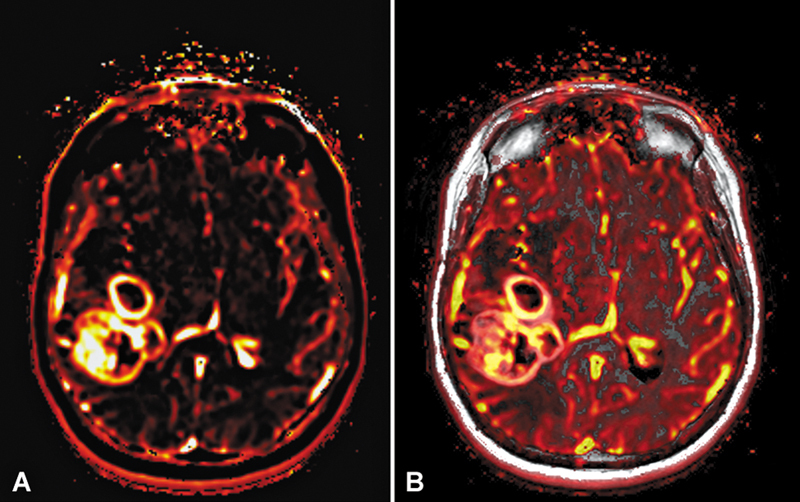
Dynamic contrast-enhanced MRI (DCE-MR), with K ^trans^ map ( **A** ) and Vp map overlaid on the T1–post-contrast image ( **B** ). Bright yellow regions represent areas of hyperperfusion, including normal vasculature and regions within the tumor, reflecting neovascularization and angiogenesis.

DCE augments the T1–post-contrast imaging by serial T1 acquisitions, providing estimation of contrast kinetics. The most widely used parameters are k ^trans^ or the efflux rate of contrast from the plasma into the extravascular extracellular space, serving as an estimate of blood–brain barrier permeability, and fractional plasma volume (Vp), which serves as a physiologic equivalent to rCBV ( Fig. 4 ). ASL is achieved by applying radiofrequency pulses to arterial blood, which invert the longitudinal magnetization of protons, effectively turning upstream arterial blood into a tracer without requiring contrast administration. Kinetic modeling enables estimation of cerebral blood flow (CBF). Similar to DSC-MRI, both DCE and ASL can help distinguish high-grade glioma from PCNSL 22 and low-grade glioma, 23 24 and both have been investigated as tools to distinguish tumor recurrence from treatment effect, 25 26 with DSC performing better than ASL and DCE in at least one study. 27 Both DSC and ASL require complex kinetic modeling, but ASL overcomes the limitations of susceptibility artifacts and eliminates the need for contrast administration required by the other techniques. Although nearly reaching routine use in brain tumor imaging, variability in perfusion acquisition sequences, contrast timing, and pre- and post-processing limits the generalizability of perfusion imaging data across institutions. 28

### Susceptibility-weighted Imaging (SWI)

**Fig. 5:**
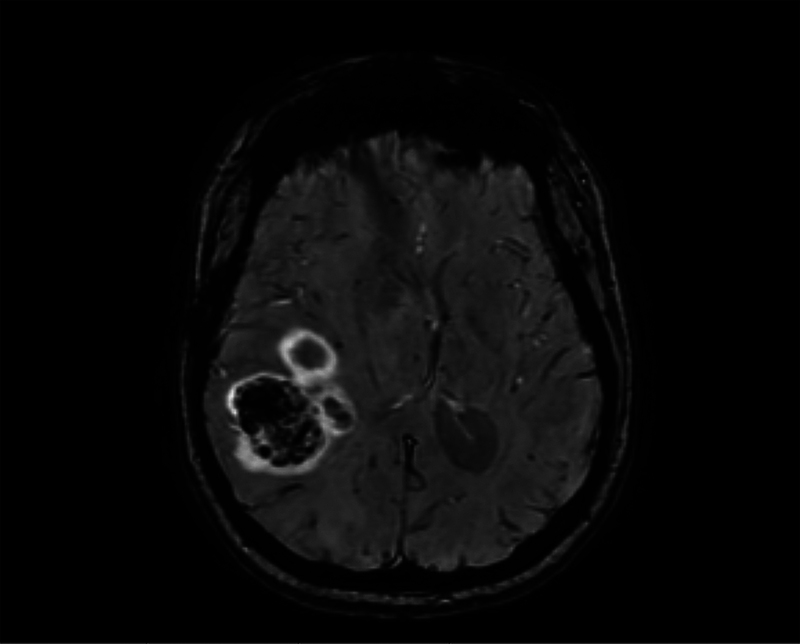
Susceptibility-weighted imaging (SWI) of a glioblastoma. The blooming artifacts within the tumor reflect the presence of intratumoral blood products, consistent with hemosiderin deposition.

Susceptibility-weighted imaging (SWI) is a technique which takes advantage of changes in the proton precession frequency revealed by T2* weighted phase signals, leading to phase differences between paramagnetic deoxygenated blood products and nearby brain parenchyma. It is particularly useful in detecting microhemorrhages, calcifications, and venous blood. 29 Increased intratumoral susceptibility signals (ITSS) have been associated with higher tumor grade, 30 as well as the ability to differentiate metastatic melanoma from breast or lung metastases 31 ( Fig. 5 ).

### Delayed Contrast

**Fig. 6:**
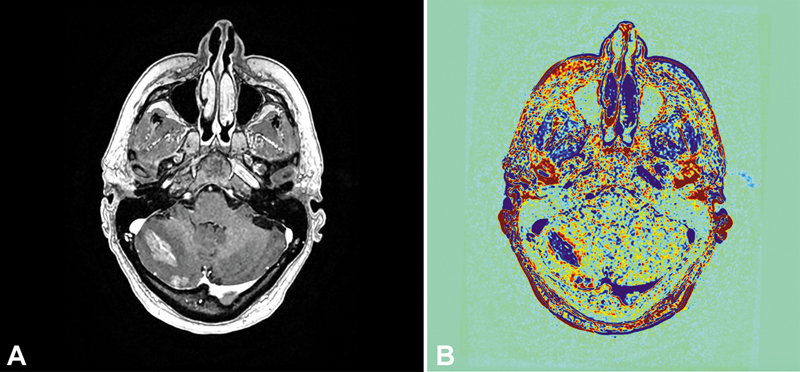
Axial T1-weighted post-contrast image of a cerebellar enhancing lesion ( **A** ) and delayed contrast-enhanced image ( **B** ). The central region of the lesion shows “blue clearance,” indicative of delayed contrast washout and suggestive of viable tumor, which was confirmed on subsequent biopsy.

Delayed contrast imaging refers to MRI sequences acquired several minutes after contrast administration, rather than immediately following injection. Timing can vary depending on the clinical context, but typically involves rescanning 5 to 15 minutes, 32 or up to 75 minutes, 33 after the initial post-contrast images. This approach can improve the detection of tumor infiltration and subtle enhancement by allowing additional time for contrast accumulation in regions with impaired blood–brain barrier integrity. It is used to help distinguish tumor progression from treatment-related effects such as radiation necrosis, as active tumor will usually have persistent enhancement due to active neovascularization and ongoing disruption of the blood–brain barrier 32 ( Fig. 6 ).

#### Diffusion Tensor

Beyond standard DWI, diffusion tensor imaging (DTI) is a technique that acquires diffusion-weighted data in at least six directions, allowing assessment of both the magnitude and direction of water diffusivity through derivation of fractional anisotropy (FA). FA ranges from 0 to 1, with 0 indicating equal diffusion in all directions and 1 indicating diffusion in a single direction. The primary application of DTI in brain tumors has been to improve the detection of tumor extent for infiltrative gliomas by combining mean diffusivity with FA in peritumoral regions. 34 35 Incorporating DTI into presurgical planning can also enhance identification of eloquent white matter tracts, aiding in functional sparing and reducing the risk of detrimental neurological deficits. 36

### Functional MRI (fMRI)

**Fig. 7:**
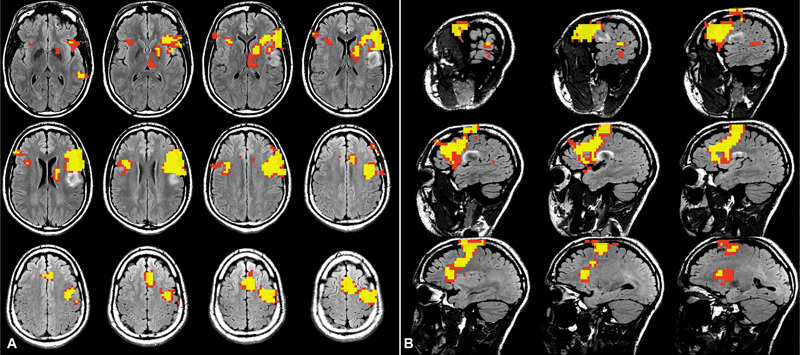
Axial ( **A** ) and sagittal ( **B** ) T2/FLAIR with fMRI overlay. Language task activation identified the T2/FLAIR hyperintense tumor at the inferior lateral-most aspect of the precentral (motor) and postcentral (sensory) gyri. The patient was determined to be left language dominant with tumor abutting Broca's area.

Functional MRI (fMRI) uses changes in blood flow as a surrogate for localized neuronal activity. The magnetic susceptibility of hemoglobin depends on its oxygenation state. While oxygenated hemoglobin is diamagnetic, deoxygenated hemoglobin is paramagnetic and exhibits a shortened T2 relaxation time. This difference generates imaging contrast termed the blood oxygen level dependent (BOLD) effect. 37 When neuronal circuits are activated, the increased demand for oxygenated blood flow leads to a relative decrease in deoxygenated blood concentration, which in turn increases the BOLD signal. Presurgical task-based fMRI is commonly used in tumor resection planning to map eloquent areas of language, visual, and motor cortex ( Fig. 7 ). When combined with traditional intraoperative direct stimulation and somatosensory evoked potentials, fMRI provides a reliable method to minimize neurologic deficit after surgery. 37 38

### Spectroscopy

Metabolic imaging is a growing field that encompasses both MR spectroscopic techniques as well as many PET tracers. Quantifying tumor metabolism, either in steady state or in response to a supply of substrate, provides biological insight far beyond anatomic imaging. Proton MR spectroscopy ( ^1^ H MRS) leverages the differential chemical shift of protons, determined by their chemical environment when subjected to a magnetic field and radiofrequency pulses. This generates a nuclear magnetic resonance (NMR) spectrum that can be spatially resolved, providing metabolic information localized to regions of interest. 39

**Fig. 8:**
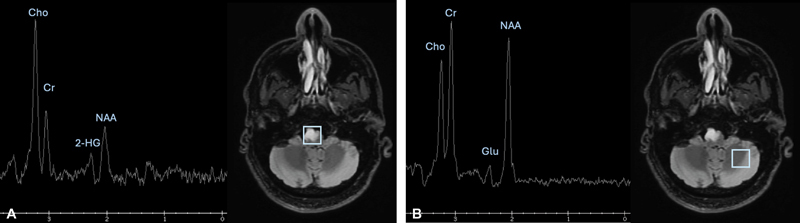
Representative single voxel ^1^ H MRS (x-axis: ppm) in nonenhancing brainstem tumor ( **A** ) and normal brain ( **B** ) demonstrating increased choline, decreased NAA, elevation of Cho/Cr (2.6; typical normal range, 0.7–1.1), elevated Cho/NAA (2.9; typical normal range 0.3–0.6), and positive 2-HG peak detected at 2.25 ppm in the tumor, supporting diagnosis of IDH-mutant low-grade glioma. 2-HG, 2-hydroxyglutarate; Cho, choline; Cr: creatine; Glu, glutamate; IDH, isocitrate dehydrogenase; MRS, magnetic resonance spectroscopy; NAA, N-acetyl aspartate; ppm, parts per million.

Single-voxel MRS acquires concentrations of metabolites in one specified area, whereas MRS Imaging (MRSI) acquires maps from multiple voxels simultaneously, generating a spatially resolved metabolite map at the expense of sensitivity and acquisition time. The most commonly analyzed metabolites in neuro-oncology are choline (Cho), lactate (lac), *N* -acetyl aspartate (NAA), glutamine-glutamate (Glx), creatine (Cr), lipids, and more recently, the oncometabolite 2-hydroxyglutarate (2-HG). Clinically, ^1^ H MRS is most often employed to differentiate between neoplastic and non-neoplastic lesions seen on MRI, particularly when surgery is not feasible or poses higher risk than standard resection or biopsy. Increased Cho and decreased NAA levels are associated with brain tumors, 40 along with a decrease in Cr 41 and increase in Glx. 42 Cho/Cr and Cho/NAA ratios have been used to predict tumor grading 43 and monitor for tumor recurrence. 44 The Lac/Glx ratio captures the characteristic metabolic reprogramming termed the Warburg Effect—the shift to anaerobic glycolysis that increases lactate production and decreases Glx generation—and has been shown to differentiate pseudoprogression from tumor recurrence. 45 Importantly, the detection of the oncometabolite 2-HG has emerged as a highly specific marker for isocitrate dehydrogenase (IDH)-mutant gliomas ( Fig. 8 ). The IDH mutation is a genetic mutation that holds significant prognostic and treatment implications in the world of primary brain tumors. 46

The interpretation of ^1^ H MRS can be limited by intratumor heterogeneity, overlap of metabolite shifts, and lack of specificity between histologies. For example, radiation necrosis and tumor progression may exhibit many overlapping metabolite concentrations, and without pre-treatment spectroscopy for comparison, the interpretation is even further limited. For these reasons, ^1^ H MRS is currently used as a complementary tool rather than a standalone diagnostic method.

Hyperpolarized MRI is an emerging technique that uses dynamic nuclear polarization to augment the signal-to-noise ratio by >10,000 fold, which, when combined with isotope labeling, enables the real-time and dynamic quantification of metabolism via MRS. 47 Using deuterium as a solvent extends the lifespan of hyperpolarized probes, enabling more clinical applications. 48 The injection of hyperpolarized [1- ^13^ C] pyruvate has demonstrated increased lactate production in brain tumor patients. 49 Ongoing studies are exploring additional labeled substrates for future clinical and research applications.

## Positron Emission Tomography (PET)

### FDG-PET

Positron emission tomography (PET) is another form of functional metabolic imaging that provides biological insight beyond what is discernable by anatomic MRI. PET takes advantage of the energy emitted from the collision of positrons emitted by decaying radiolabeled isotopes and nearby electrons. The resulting photons are absorbed by the scintillation crystals in PET cameras, producing light that is converted into an electrical signal. 50

^18^ F-2-fluoro-2-deoxy-D-glucose ( ^18^ F-FDG) is a glucose analog and the most widely used PET tracer in clinical nuclear medicine, with numerous applications in oncology. Many cancers are characterized by an increase in anaerobic glycolysis (Warburg effect), driven by overexpression of glucose transporters. 51 As a result, FDG is preferentially taken up by malignant cells. Upon transport into the cell, ^18^ F-FDG is phosphorylated and, in the absence of high glucose-6-phosphatase activity, becomes effectively trapped, unable to undergo further metabolism. This leads to accumulation in malignant and other metabolically active cells that upregulate glycolysis.

**Fig. 9:**
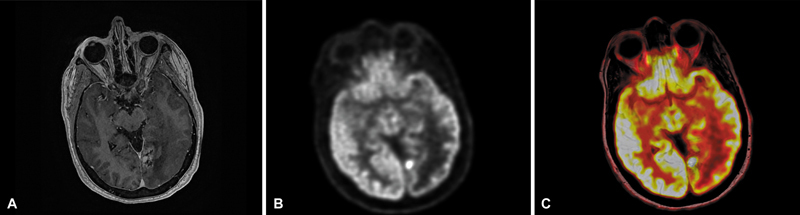
Axial T1-weighted post-contrast image of a left occipital lesion ( **A** ) that had previously received radiation. FDG-PET MR showed a focal area of hypermetabolic activity ( **B, C** ). Subsequent resection confirmed active tumor amid radiation necrosis.

Historically, ^18^ F-FDG-PET has been used in neuro-oncology for distinguishing high-grade gliomas from other malignant brain tumors, delineating tumor “hot spots” to guide biopsy, 52 differentiating between radiation necrosis and active tumor, 53 and serial imaging to assess treatment response 54 ( Fig. 9 ). In glioblastoma, increased ^18^ F-FDG-PET uptake has been shown to correlate with decreased survival in both the newly diagnosed 55 56 and post-treatment setting. 57 58 In CNS lymphoma, a systematic review estimated the pooled diagnostic sensitivity of ^18^ F-FDG-PET at 0.88 (95% CI: 0.80–0.94) and specificity at 0.86 (95% CI: 0.73–0.94). 59 Furthermore, multiple studies have established prognostic value from pre-treatment ^18^ F-FDG-PET scans in this population. 60 61 Additionally, ^18^ F-FDG-PET can help distinguish glioblastoma from CNS lymphoma, 52 62 increasing the diagnostic value in this population.

Despite these informative uses, there are many limitations of ^18^ F-FDG-PET in the management of brain tumors. Although ^18^ F-FDG-PETcan be used to assess brain metastasis, it does not perform as well as contrast-enhanced MRI. Studies evaluating its role in differentiating radiation treatment effects from viable tumor in brain metastasis are limited by their small sample sizes, heterogeneous histologies, and variable SUV cutoffs, leading to pooled sensitivity and specificity estimates of 40 to 95% and 50 to 100%, respectively. 63 High background uptake in the cortex and basal ganglia severely limits the detection of hypermetabolic lesions in these areas. Additionally, although ^18^ F-FDG-PET is generally considered to be a sensitive measure of hypermetabolism, it does not achieve the specificity needed to distinguish high-grade gliomas from secondary metastatic tumors, nor can it differentiate brain abscesses, fungal lesions, granulomatous disease, or tumefactive demyelinating lesions. 64 For these reasons, PET applications in the management of primary and secondary brain tumors are shifting to alternative probes, such as amino acid tracers. A 2019 consensus summarized by the Response Assessment in Neuro-Oncology(RANO)/PET PET RANO working group concluded that in 2019, the utility of ^18^ F-FDG-PET in brain metastasis is outperformed by amino acid PET tracers for every indication. 63

### Amino Acid PET

Multiple primary research studies and consensus guidelines have outlined the superiority of amino acid PET tracers in the management of patients with primary and secondary brain tumors. 4 63 65 66 The most used amino acid PET tracers are *O* -(2- ^18^ F-fluoroethyl)-l-tyrosine ( ^18^ F-FET), ^11^ C-methyl-l-methionine ( ^11^ C-MET), and 3,4-dihydroxy-6- ^18^ F-fluoro-l-phenylalanine ( ^18^ F-FDOPA). Two subtypes of L-type large neutral amino acid transporters (LAT1 and LAT2) are overexpressed in gliomas and brain metastasis, facilitating uptake of these tracers. 67 68 Anti-1-amino-3- ^18^ F-fluorocyclobutane-1-carboxylic acid ( ^18^ F-fluciclovine) is a synthetic amino acid analog that has gained interest in glioma and is predominantly transported by the neutral alanine, serine, and cysteine transporter 2 in addition to LAT1. 69 The relatively low physiologic uptake of amino acid tracers in normal brain tissue enables high contrast between tumor and surrounding unaffected tissue and offers a distinct advantage over ^18^ F-FDG. Although extensively used in Europe, limited availability remains the biggest barrier to widespread use of amino acid PET tracers in the United States.

Amino acid PET, most frequently using ^18^ F-FET, has been evaluated in many of the same applications as ^18^ F-FDG. A meta-analysis of 462 patients demonstrated superior diagnostic accuracy of ^18^ F-FET over ^18^ F-FDG for identifying primary brain tumors, 70 although some reports have noted positive uptake in brain abscesses and demyelinating lesions. 71 72 While differentiating between higher and lower grade gliomas remains a challenge in both amino acid and glucose analogs, ^11^ C-MET, ^18^ F-FET, and ^18^ F-FDOPA PET all outperform standard MRI in delineating tumor extent in both contrast-enhancing and nonenhancing tumors. 73 74 75 76 77 78 79 80 This information can aid surgical planning, particularly for selecting biopsy sites within heterogeneously enhancing lesions or when tumor is abutting eloquent functional brain areas. 81 82 83

Although studies have incorporated amino acid PETs into radiation planning workflows, they have not yet demonstrated a survival benefit. 84 However, the prognostic value of amino acid PET has been demonstrated in many studies of all grades of glioma using both static and dynamic-derived parameters. 85 86 87 88 In grade 3 and 4 tumors, multiple studies have shown that a reduction in amino acid uptake or decrease in tumor volume measured by amino acid PET indicates treatment response and correlates with improved outcomes. 89 90 91 92 93 Additionally, amino acid PET has outperformed MRI in assessing treatment response to antiangiogenic therapy with bevacizumab. 94 95 96 97

Importantly, amino acid PET demonstrates utility in differentiating treatment effects from progressive disease in both glioma and brain metastasis. 79 98 99 100 In the latter, the limited available data show high diagnostic accuracy of amino acid over ^18^ F-FDG-PET in diagnosis, treatment response, and differentiating immunotherapy-induced changes. 63

### Somatostatin Receptor Analog PET

**Fig. 10:**
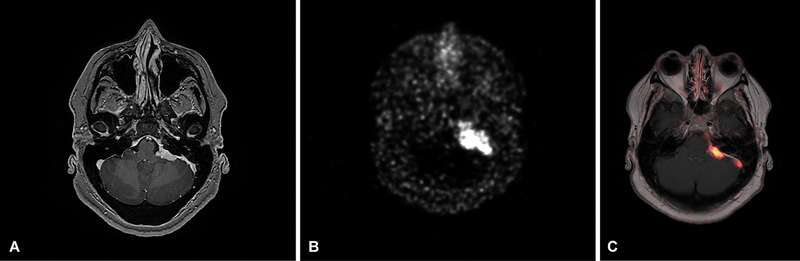
Axial T1-weighted post-contrast image of a left posterior fossa extra-axial dural-based lesion ( **A** ). [ ^68^ Ga]Ga DOTATATE PET shows intense tracer avidity of the lesion ( **B, C** ), supporting the diagnosis of meningioma.

There are several ^68^ Ga-labeled DOTA chelator-conjugated somatostatin analogs that bind with high affinity to somatostatin receptors, including [ ^68^ Ga]Ga-DOTA-D-Phe1-Tyr3-octreotide ([ ^68^ Ga]Ga-DOTATOC), [ ^68^ Ga]Ga-DOTA-D-Phe1-Tyr3-octreotate ([ ^68^ Ga]Ga-DOTATATE), and [ ^68^ Ga]Ga-DOTA-D-Phe1-Nal3-octreotide ([ ^68^ Ga]Ga-DOTANOC). 101 Although [ ^68^ Ga]Ga-DOTA-SSTR PET was initially developed for imaging pituitary adenomas, the overexpression of somatostatin subtype receptor 2 (SSTR2) on meningiomas has enabled its application in these tumor types as well 102 ( Fig. 10 ).

Given the high specificity of the SSTR2 receptor to these tumors when compared with normal brain, [ ^68^ Ga]Ga-DOTA-SSTR PET has demonstrated high diagnostic accuracy in identifying meningioma, 103 104 evaluation of infracranial/transosseous extent of tumor, 103 and differentiating from dural-based brain metastasis. 105 106 The use of [ ^68^ Ga]Ga-DOTA-SSTR PET for contouring tumor volume to guide radiation therapy planning has also shown promise in both meningioma 107 108 and in pituitary carcinoma invading the cavernous sinus. 109 The combination of ^18^ F-FDG with [ ^68^ Ga]Ga-DOTA has been shown to identify residual pituitary adenoma after resection, 110 and the detection of both primary and secondary pituitary carcinomas is highly accurate. 111 112 However, due to the limited number of available research studies, SSTR analogs remain an adjunct tool in the management of meningioma and pituitary adenomas or carcinomas.

## Clinical Decision Frameworks

Standardized imaging criteria are essential for evaluating tumor response, guiding clinical decisions, and supporting enrollment and assessment in clinical trials. One of the most widely used frameworks in neuro-oncology is the Response Assessment in Neuro-Oncology (RANO) criteria, designed to improve consistency in evaluating patients with brain tumors, especially in clinical trial settings. 113

The original RANO criteria, introduced in 2010, were developed to address limitations of the earlier Macdonald criteria, which relied solely on changes in contrast-enhancing tumor on T1-weighted MRI. 114 115 RANO criteria incorporate nonenhancing disease on T2/FLAIR, neurologic status, and corticosteroid use, offering a more comprehensive and clinically relevant assessment ( Table 1 ). These criteria apply most commonly to high-grade gliomas but have also been adapted for other tumor types. RANO-BM (Response Assessment in Neuro-Oncology for Brain Metastases) offers guidance on assessing intracranial metastatic disease, accounting for the number, size, and location of lesions, as well as the presence of extracranial disease. 116 RANO-BM allows for the selection and longitudinal measurement of up to five individual CNS target lesions, enabling lesion-specific tracking. This approach is necessary in patients with multiple brain metastases, where responses may vary across lesions. RANO-LM, designed for leptomeningeal disease, emphasizes radiographic features such as linear versus nodular enhancement on MRI, in combination with neurologic symptoms and cerebrospinal fluid cytology, to assess progression and response. 117

**Table 1:** Response Assessment in Neuro-Oncology (RANO) 2.0 criteria for enhancing tumors

	Imaging features	Clinical features
Complete response	● Disappearance of all enhancing disease (measurable and non-measurable) ● Sustained for at least 4 weeks ● No new lesions	● No corticosteroids (physiological replacement doses allowed) ● Clinically stable or improved
Partial response	● ≥65% decrease in volumetric measurement of enhancing tumor compared with baseline or ≥50% decrease in the product of perpendicular diameters ● Sustained for at least 4 weeks ● No progression of non-measurable disease ● No new lesions	● Stable or reduced corticosteroids (compared with baseline) ● Clinically stable or improved
Stable disease	● Does not qualify for complete response, partial response, or progression ● Stable area(s) of enhancing target lesions on imaging ● No new lesions ● No progression of non-measurable disease or nontarget lesions	● Stable or reduced corticosteroids (compared with baseline) ● Clinically stable
Progression	● ≥40% increase in volumetric measurement of enhancing tumor or a ≥25% increase in the product of perpendicular diameters, on stable or increasing steroid dose ● Clear progression of nonmeasurable lesions ● Any new lesion measuring ≥10 mm × 10 mm in perpendicular diameters ● Appearance of definite leptomeningeal disease ● Unequivocal progression of existing nontarget lesions	● Clinical deterioration (not attributable to other non-tumor causes and not due to steroid decrease) ● Failure to return for evaluation because of death or deteriorating condition should also be considered as progression unless caused by documented nonrelated disorders

Note: This framework updates prior RANO guidelines by incorporating volumetric measurements, emphasizing confirmation of progression, and refining criteria for T2/FLAIR progression to improve diagnostic consistency in clinical trials and routine practice. 140

For patients receiving immunotherapy, iRANO (Immunotherapy Response Assessment in Neuro-Oncology) was developed to address the phenomenon of pseudoprogression, where immune infiltration can transiently enlarge lesions or cause new enhancement. 118 iRANO recommends deferring a formal declaration of progression within the first 6 months of immunotherapy unless there is unequivocal clinical decline, helping prevent the premature discontinuation of potentially effective treatment.

In spinal cord tumors, no formal RANO criteria exist, but imaging assessments follow similar principles, using contrast-enhanced and T2-weighted spinal MRI to evaluate cord expansion, enhancement patterns, and signal changes over time. For meningiomas, volumetric assessment on MRI and serial evaluation of contrast enhancement remain standard, and ^68^ Ga-DOTATATE PET is occasionally used. 119 Although typically slow-growing, meningiomas can recur or progress unpredictably, particularly in higher grade subtypes or after subtotal resection.

The PET Response Assessment in Neuro-Oncology (PET-RANO) group has recently proposed consensus guidelines to integrate PET imaging into standardized response criteria for gliomas. 66 Recognizing the limitations of MRI alone in differentiating true progression from treatment-related effects, PET-RANO incorporates metabolic imaging findings, primarily from amino acid PET, into response assessments alongside conventional MRI, neurologic status, and steroid use. This combined approach aims to improve diagnostic accuracy in challenging scenarios such as pseudoprogression, post-radiation changes, and evaluation of nonenhancing tumor burden. Although PET-RANO criteria are still in early implementation phases, they represent an important step toward harmonizing multimodal imaging interpretation in clinical trials and practice.

In 2023, the RANO 2.0 working group proposed updated response criteria for gliomas, reflecting changes in clinical practice and the increasing use of molecular classification 120 ( Table 1 ). RANO 2.0 retains the core principles of the original framework but integrates more consistent guidelines for imaging timing, standardized volumetric thresholds, and decision rules that incorporate IDH status and other biomarkers. It also emphasizes harmonization with clinical trial design, ensuring that response assessments align with therapeutic mechanisms and endpoints.

Together, these frameworks reflect the growing complexity of neuro-oncologic care and the need for disease- and treatment-specific imaging strategies. For neurologists, familiarity with these criteria enhances the ability to interpret imaging meaningfully and to guide patients through increasingly nuanced treatment pathways.

## Emerging Frontiers

Radiomics uses artificial intelligence (AI) to extract features from routine imaging surveillance that cannot be detected through conventional imaging analysis. Images typically undergo pre-processing, tumor segmentation, and feature extraction, after which a machine-learning model is generated and validated. 121 These techniques have been applied to MRI, 122 amino acid PET, 123 and MRS. 124 Several studies have demonstrated potential utility in glioma and brain metastasis, including identifying biomarkers, 125 predicting survival, 122 and distinguishing treatment effects from viable tumor. 126 127 These techniques are being increasingly incorporated into clinical trials. 128 Radiogenomics combines available genomic data to a radiomics analysis, thereby increasing the accuracy of machine learning algorithms. 122 Limitations include a lack of standardization in evaluating and reporting high-quality radiomics in studies, a missing emphasis on the biologic meaning of radiomics features, and the absence of multicenter validation. 129

The development of imaging tracers, both targeting specific receptors and interrogating metabolic substrates beyond pyruvate, has increased rapidly over recent decades. These include PET tracers targeting epidermal growth factor receptor (EGFR) expression in gliomas, chemokine receptor type 4 (CXCR4) in lymphoma, translocator protein (TSPO), and prostate-specific membrane antigen (PSMA). 130 Imaging of hypoxia and other amino acids are growing applications, and PET probes targeting the immune system, specifically the metabolic reprogramming of T-cells, 130 represent an exciting frontier in the age of immunotherapy. Hyperpolarized MRS probes under study include [2- ^13^ C] pyruvate, 131 [1- ^13^ C]α-ketoglutarate, and [1- ^13^ C]glutamate in IDH-mutant gliomas, 132 [1- ^13^ C] urea for brain perfusion, 133 and [5- ^13^ C,4,4- ^2^ H2,5- ^15^ N]-L-glutamine to monitor glutamine metabolism. 134 The combination of [1- ^13^ C] dehydroascorbic acid with [1- ^13^ C] pyruvate has demonstrated the ability to probe redox status and glycolytic flux simultaneously, 135 with promising neurologic and oncologic applications.

## Equity in Access

Despite advances in neuro-oncologic imaging, access to high-resolution MRI, MR perfusion, spectroscopy, and PET imaging remains uneven across healthcare settings. 136 Patients treated in safety-net hospitals, rural centers, or under public insurance plans may face limited availability of these tools, delayed scheduling, or outright denial of coverage, particularly for sequences considered non-standard despite their clinical utility. 137 These disparities can lead to delayed diagnoses, ambiguous treatment-response assessments, and reduced eligibility for clinical trials that require advanced imaging. 138 Furthermore, lack of access to centralized imaging review or volumetric tools can undermine consistency in care. Addressing these gaps requires broader policy efforts, including reimbursement reform, infrastructure investment in underserved areas, and inclusion of equity metrics in trial design. 139 As imaging becomes increasingly central to precision neuro-oncology, ensuring equitable access must remain a core priority.

## Conclusion

Neuroimaging plays a central role in the diagnosis, management, and surveillance of patients with primary and metastatic brain tumors. Advances in MRI techniques—including perfusion imaging, diffusion tensor imaging, functional MRI, susceptibility-weighted imaging, and MR spectroscopy—as well as complementary modalities such as amino acid PET, have significantly enhanced our ability to characterize tumor biology, assess treatment response, and guide surgical and radiation planning. Standardized frameworks such as the RANO criteria have brought greater consistency to clinical trial assessment and routine care, though interpretation remains nuanced due to phenomena like pseudoprogression, pseudoresponse, and treatment-related effects. Emerging applications of radiomics and artificial intelligence hold promise for augmenting imaging interpretation by extracting quantitative features and predictive biomarkers not readily discernible through conventional analysis. As imaging continues to evolve alongside molecular classification and targeted therapies, it is critical to ensure equitable access to advanced modalities across healthcare settings. For neurologists and other clinicians, a working knowledge of these imaging tools and clinical decision frameworks is essential to delivering precise, patient-centered neuro-oncologic care.

## References

[1] Price M Ballard C Benedetti J CBTRUS statistical report: primary brain and other central nervous system tumors diagnosed in the United States in 2017-2021 Neuro-oncol 2024 26 06 vi1 vi85 39371035 10.1093/neuonc/noae145 PMC11456825

[2] Nayak L Lee E Q Wen P Y Epidemiology of brain metastases Curr Oncol Rep 2012 14 01 48 54 22012633 10.1007/s11912-011-0203-y

[3] Ostrom Q T Wright C H Barnholtz-Sloan J S Brain metastases: epidemiology Handb Clin Neurol 2018 149 27 42 29307358 10.1016/B978-0-12-811161-1.00002-5

[4] Langen K J Galldiks N Hattingen E Shah N J Advances in neuro-oncology imaging Nat Rev Neurol 2017 13 05 279 289 28387340 10.1038/nrneurol.2017.44

[5] Carl B Bopp M Saß B Reliable navigation registration in cranial and spine surgery based on intraoperative computed tomography Neurosurg Focus 2019 47 06 E11 10.3171/2019.8.FOCUS19621 31786552

[6] Wang M Song Z Guidelines for the placement of fiducial points in image-guided neurosurgery Int J Med Robot 2010 6 02 142 149 20131341 10.1002/rcs.299

[7] Jumpstarting Brain Tumor Drug Development Coalition Imaging Standardization Steering Committee Ellingson B M Bendszus M Boxerman J Consensus recommendations for a standardized Brain Tumor Imaging Protocol in clinical trials Neuro-oncol 2015 17 09 1188 1198 26250565 10.1093/neuonc/nov095 PMC4588759

[8] Ellingson B M Wen P Y Cloughesy T F Evidence and context of use for contrast enhancement as a surrogate of disease burden and treatment response in malignant glioma Neuro-oncol 2018 20 04 457 471 29040703 10.1093/neuonc/nox193 PMC5909663

[9] Meier R Knecht U Loosli T Clinical evaluation of a fully-automatic segmentation method for longitudinal brain tumor volumetry Sci Rep 2016 6 23376 27001047 10.1038/srep23376 PMC4802217

[10] Peiris H Hayat M Chen Z Egan G Harandi M A robust volumetric transformer for accurate 3D tumor segmentation Springer Nature Switzerland 2022 162 172

[11] Rajput S Kapdi R Roy M Raval M S A triplanar ensemble model for brain tumor segmentation with volumetric multiparametric magnetic resonance images Healthc Anal (N Y) 2024 5 100307

[12] van den Bent M J Cloughesy T F Ellingson B M The use of minor response, volumetric assessment, and growth rate kinetics as endpoints in grade 1–3 glioma clinical trials: a RANO perspective Neuro-oncol 2025 noaf173.Epub ahead of print 10.1093/neuonc/noaf173 40692474 PMC12833540

[13] Wen P Y Macdonald D R Reardon D A Updated response assessment criteria for high-grade gliomas: response assessment in neuro-oncology working group J Clin Oncol 2010 28 11 1963 1972 20231676 10.1200/JCO.2009.26.3541

[14] Hattingen E Jurcoane A Daneshvar K Quantitative T2 mapping of recurrent glioblastoma under bevacizumab improves monitoring for non-enhancing tumor progression and predicts overall survival Neuro-oncol 2013 15 10 1395 1404 23925453 10.1093/neuonc/not105 PMC3779046

[15] Guo A C Cummings T J Dash R C Provenzale J M Lymphomas and high-grade astrocytomas: comparison of water diffusibility and histologic characteristics Radiology 2002 224 01 177 183 12091680 10.1148/radiol.2241010637

[16] Zulfiqar M Yousem D M Lai H ADC values and prognosis of malignant astrocytomas: does lower ADC predict a worse prognosis independent of grade of tumor?—a meta-analysis AJR Am J Roentgenol 2013 200 03 624 629 23436853 10.2214/AJR.12.8679

[17] van Dijken B RJ van Laar P J Holtman G A van der Hoorn A Diagnostic accuracy of magnetic resonance imaging techniques for treatment response evaluation in patients with high-grade glioma, a systematic review and meta-analysis Eur Radiol 2017 27 10 4129 4144 28332014 10.1007/s00330-017-4789-9 PMC5579204

[18] Yu Y Ma Y Sun M Jiang W Yuan T Tong D Meta-analysis of the diagnostic performance of diffusion magnetic resonance imaging with apparent diffusion coefficient measurements for differentiating glioma recurrence from pseudoprogression Medicine (Baltimore) 2020 99 23 e20270 32501974 10.1097/MD.0000000000020270 PMC7306328

[19] Padhani A R Liu G Koh D M Diffusion-weighted magnetic resonance imaging as a cancer biomarker: consensus and recommendations Neoplasia 2009 11 02 102 125 19186405 10.1593/neo.81328 PMC2631136

[20] Suh C H Kim H S Jung S C Park J E Choi C G Kim S J MRI as a diagnostic biomarker for differentiating primary central nervous system lymphoma from glioblastoma: a systematic review and meta-analysis J Magn Reson Imaging 2019 50 02 560 572 30637843 10.1002/jmri.26602

[21] Suh C H Kim H S Jung S C Choi C G Kim S J Perfusion MRI as a diagnostic biomarker for differentiating glioma from brain metastasis: a systematic review and meta-analysis Eur Radiol 2018 28 09 3819 3831 29619517 10.1007/s00330-018-5335-0

[22] Xu W Wang Q Shao A Xu B Zhang J The performance of MR perfusion-weighted imaging for the differentiation of high-grade glioma from primary central nervous system lymphoma: a systematic review and meta-analysis PLoS One 2017 12 03 e0173430 28301491 10.1371/journal.pone.0173430 PMC5354292

[23] Okuchi S Rojas-Garcia A Ulyte A Diagnostic accuracy of dynamic contrast-enhanced perfusion MRI in stratifying gliomas: a systematic review and meta-analysis Cancer Med 2019 8 12 5564 5573 31389669 10.1002/cam4.2369 PMC6745862

[24] Falk Delgado A De Luca F van Westen D Falk Delgado A Arterial spin labeling MR imaging for differentiation between high- and low-grade glioma-a meta-analysis Neuro-oncol 2018 20 11 1450 1461 29868920 10.1093/neuonc/noy095 PMC6176798

[25] Patel P Baradaran H Delgado D MR perfusion-weighted imaging in the evaluation of high-grade gliomas after treatment: a systematic review and meta-analysis Neuro-oncol 2017 19 01 118 127 27502247 10.1093/neuonc/now148 PMC5193025

[26] Wan B Wang S Tu M Wu B Han P Xu H The diagnostic performance of perfusion MRI for differentiating glioma recurrence from pseudoprogression: a meta-analysis Medicine (Baltimore) 2017 96 11 e6333 28296759 10.1097/MD.0000000000006333 PMC5369914

[27] Zhang J Wang Y Wang Y Perfusion magnetic resonance imaging in the differentiation between glioma recurrence and pseudoprogression: a systematic review, meta-analysis and meta-regression Quant Imaging Med Surg 2022 12 10 4805 4822 36185045 10.21037/qims-22-32 PMC9511424

[28] Galldiks N Kaufmann T J Vollmuth P Challenges, limitations, and pitfalls of PET and advanced MRI in patients with brain tumors: a report of the PET/RANO group Neuro-oncol 2024 26 07 1181 1194 38466087 10.1093/neuonc/noae049 PMC11226881

[29] Mittal S Wu Z Neelavalli J Haacke E M Susceptibility-weighted imaging: technical aspects and clinical applications, part 2 AJNR Am J Neuroradiol 2009 30 02 232 252 19131406 10.3174/ajnr.A1461 PMC3805373

[30] Li X Zhu Y Kang H Glioma grading by microvascular permeability parameters derived from dynamic contrast-enhanced MRI and intratumoral susceptibility signal on susceptibility weighted imaging Cancer Imaging 2015 15 01 4 25889239 10.1186/s40644-015-0039-z PMC4389664

[31] Schwarz D Bendszus M Breckwoldt M O Clinical value of susceptibility weighted imaging of brain metastases Front Neurol 2020 11 55 32117017 10.3389/fneur.2020.00055 PMC7010951

[32] Kushnirsky M Nguyen V Katz J S Time-delayed contrast-enhanced MRI improves detection of brain metastases and apparent treatment volumes J Neurosurg 2016 124 02 489 495 26361281 10.3171/2015.2.JNS141993

[33] Satvat N Korczynski O Müller-Eschner M A rapid late enhancement MRI protocol improves differentiation between brain tumor recurrence and treatment-related contrast enhancement of brain parenchyma Cancers (Basel) 2022 14 22 5523 36428617 10.3390/cancers14225523 PMC9688406

[34] Jiang R Du F Z He C Gu M Ke Z W Li J H The value of diffusion tensor imaging in differentiating high-grade gliomas from brain metastases: a systematic review and meta-analysis PLoS One 2014 9 11 e112550 25380185 10.1371/journal.pone.0112550 PMC4224505

[35] Miloushev V Z Chow D S Filippi C G Meta-analysis of diffusion metrics for the prediction of tumor grade in gliomas AJNR Am J Neuroradiol 2015 36 02 302 308 25190201 10.3174/ajnr.A4097 PMC7965672

[36] Manan A A Yahya N Idris Z Manan H A The utilization of diffusion tensor imaging as an image-guided tool in brain tumor resection surgery: a systematic review Cancers (Basel) 2022 14 10 2466 35626069 10.3390/cancers14102466 PMC9139820

[37] Bogomolny D L Petrovich N M Hou B L Peck K K Kim M JJ Holodny A I Functional MRI in the brain tumor patient Top Magn Reson Imaging 2004 15 05 325 335 15627006 10.1097/00002142-200410000-00005

[38] Stopa B M Senders J T Broekman M LD Vangel M Golby A J Preoperative functional MRI use in neurooncology patients: a clinician survey Neurosurg Focus 2020 48 02 E11 10.3171/2019.11.FOCUS19779 PMC7818717 32006949

[39] Zhu H Barker P B MR spectroscopy and spectroscopic imaging of the brain Methods Mol Biol 2011 711 203 226 21279603 10.1007/978-1-61737-992-5_9 PMC3416028

[40] Dhermain F G Hau P Lanfermann H Jacobs A H van den Bent M J Advanced MRI and PET imaging for assessment of treatment response in patients with gliomas Lancet Neurol 2010 9 09 906 920 20705518 10.1016/S1474-4422(10)70181-2

[41] Bulik M Jancalek R Vanicek J Skoch A Mechl M Potential of MR spectroscopy for assessment of glioma grading Clin Neurol Neurosurg 2013 115 02 146 153 23237636 10.1016/j.clineuro.2012.11.002

[42] Ekici S Nye J A Neill S G Allen J W Shu H-K Fleischer C C Glutamine imaging: a new avenue for glioma management AJNR Am J Neuroradiol 2022 43 01 11 18 34737183 10.3174/ajnr.A7333 PMC8757564

[43] Rafique Z Awan M W Iqbal S Diagnostic accuracy of magnetic resonance spectroscopy in predicting the grade of glioma keeping histopathology as the gold standard Cureus 2022 14 02 e22056 35340513 10.7759/cureus.22056 PMC8916061

[44] Aseel A McCarthy P Mohammed A Brain magnetic resonance spectroscopy to differentiate recurrent neoplasm from radiation necrosis: a systematic review and meta-analysis J Neuroimaging 2023 33 02 189 201 36631883 10.1111/jon.13080

[45] El-Abtah M E Talati P Fu M Magnetic resonance spectroscopy outperforms perfusion in distinguishing between pseudoprogression and disease progression in patients with glioblastoma Neurooncol Adv 2022 4 01 vdac128 36071927 10.1093/noajnl/vdac128 PMC9446677

[46] Choi C Ganji S K DeBerardinis R J 2-hydroxyglutarate detection by magnetic resonance spectroscopy in IDH-mutated patients with gliomas Nat Med 2012 18 04 624 629 22281806 10.1038/nm.2682 PMC3615719

[47] Jørgensen S H Bøgh N Hansen E Væggemose M Wiggers H Laustsen C Hyperpolarized MRI—an update and future perspectives Semin Nucl Med 2022 52 03 374 381 34785033 10.1053/j.semnuclmed.2021.09.001

[48] Deh K Zhang G Park A H First in-human evaluation of [1- ^13^ C]pyruvate in D _2_ O for hyperpolarized MRI of the brain: a safety and feasibility study Magn Reson Med 2024 91 06 2559 2567 38205934 10.1002/mrm.30002 PMC11009889

[49] Miloushev V Z Granlund K L Boltyanskiy R Metabolic imaging of the human brain with hyperpolarized ^13^ C pyruvate demonstrates ^13^ C lactate production in brain tumor patients Cancer Res 2018 78 14 3755 3760 29769199 10.1158/0008-5472.CAN-18-0221 PMC6050093

[50] Berger A How does it work? Positron emission tomography BMJ 2003 326 7404 1449 12829560 10.1136/bmj.326.7404.1449 PMC1126321

[51] Liberti M V Locasale J W The Warburg effect: how does it benefit cancer cells? Trends Biochem Sci 2016 41 03 211 218 26778478 10.1016/j.tibs.2015.12.001 PMC4783224

[52] Verger A Langen K J PET imaging in glioblastoma: use in clinical practice Codon Publications 2017. Accessed August 1, 2025 at:http://www.ncbi.nlm.nih.gov/books/NBK469986/29251869

[53] Ninatti G Pini C Gelardi F Sollini M Chiti A The role of PET imaging in the differential diagnosis between radiation necrosis and recurrent disease in irradiated adult-type diffuse gliomas: a systematic review Cancers (Basel) 2023 15 02 364 36672314 10.3390/cancers15020364 PMC9856914

[54] Spence A M Muzi M Graham M M 2-[(18)F]Fluoro-2-deoxyglucose and glucose uptake in malignant gliomas before and after radiotherapy: correlation with outcome Clin Cancer Res 2002 8 04 971 979 11948102

[55] De Witte O Lefranc F Levivier M Salmon I Brotchi J Goldman S FDG-PET as a prognostic factor in high-grade astrocytoma J Neurooncol 2000 49 02 157 163 11206011 10.1023/a:1026518002800

[56] Colavolpe C Metellus P Mancini J Independent prognostic value of pre-treatment 18-FDG-PET in high-grade gliomas J Neurooncol 2012 107 03 527 535 22169956 10.1007/s11060-011-0771-6

[57] Bai J W Qiu S Q Zhang G J Molecular and functional imaging in cancer-targeted therapy: current applications and future directions Signal Transduct Target Ther 2023 8 01 89 36849435 10.1038/s41392-023-01366-y PMC9971190

[58] Omuro A Beal K Gutin P Phase II study of bevacizumab, temozolomide, and hypofractionated stereotactic radiotherapy for newly diagnosed glioblastoma Clin Cancer Res 2014 20 19 5023 5031 25107913 10.1158/1078-0432.CCR-14-0822 PMC4523080

[59] Zou Y Tong J Leng H Jiang J Pan M Chen Z Diagnostic value of using 18F-FDG PET and PET/CT in immunocompetent patients with primary central nervous system lymphoma: a systematic review and meta-analysis Oncotarget 2017 8 25 41518 41528 28514747 10.18632/oncotarget.17456 PMC5522282

[60] Krebs S Mauguen A Yildirim O Prognostic value of [ ^18^ F]FDG PET/CT in patients with CNS lymphoma receiving ibrutinib-based therapies Eur J Nucl Med Mol Imaging 2021 48 12 3940 3950 33966087 10.1007/s00259-021-05386-0 PMC8484020

[61] Albano D Bertoli M Battistotti M Prognostic role of pretreatment 18F-FDG PET/CT in primary brain lymphoma Ann Nucl Med 2018 32 08 532 541 29982990 10.1007/s12149-018-1274-8

[62] Kosaka N Tsuchida T Uematsu H Kimura H Okazawa H Itoh H 18F-FDG PET of common enhancing malignant brain tumors AJR Am J Roentgenol 2008 190 06 W365-9 18492879 10.2214/AJR.07.2660

[63] Galldiks N Langen K J Albert N L PET imaging in patients with brain metastasis-report of the RANO/PET group Neuro-oncol 2019 21 05 585 595 30615138 10.1093/neuonc/noz003 PMC6502495

[64] Omuro A M Leite C C Mokhtari K Delattre J Y Pitfalls in the diagnosis of brain tumours Lancet Neurol 2006 5 11 937 948 17052661 10.1016/S1474-4422(06)70597-X

[65] Albert N L Weller M Suchorska B Response Assessment in Neuro-Oncology working group and European Association for Neuro-Oncology recommendations for the clinical use of PET imaging in gliomas Neuro-oncol 2016 18 09 1199 1208 27106405 10.1093/neuonc/now058 PMC4999003

[66] Albert N L Galldiks N Ellingson B M PET-based response assessment criteria for diffuse gliomas (PET RANO 1.0): a report of the RANO group Lancet Oncol 2024 25 01 e29 e41 38181810 10.1016/S1470-2045(23)00525-9 PMC11787868

[67] Wiriyasermkul P Nagamori S Tominaga H Transport of 3-fluoro-L-α-methyl-tyrosine by tumor-upregulated L-type amino acid transporter 1: a cause of the tumor uptake in PET J Nucl Med 2012 53 08 1253 1261 22743251 10.2967/jnumed.112.103069

[68] Papin-Michault C Bonnetaud C Dufour M Study of LAT1 expression in brain metastases: towards a better understanding of the results of positron emission tomography using amino acid tracers PLoS One 2016 11 06 e0157139 27276226 10.1371/journal.pone.0157139 PMC4898730

[69] Ono M Oka S Okudaira H Comparative evaluation of transport mechanisms of trans-1-amino-3-[ ^18^ F]fluorocyclobutanecarboxylic acid and L-[methyl- ^11^ C]methionine in human glioma cell lines Brain Res 2013 1535 24 37 23994214 10.1016/j.brainres.2013.08.037

[70] Dunet V Rossier C Buck A Stupp R Prior J O Performance of 18F-fluoro-ethyl-tyrosine (18F-FET) PET for the differential diagnosis of primary brain tumor: a systematic review and Metaanalysis J Nucl Med 2012 53 02 207 214 22302961 10.2967/jnumed.111.096859

[71] Floeth F W Pauleit D Sabel M 18F-FET PET differentiation of ring-enhancing brain lesions J Nucl Med 2006 47 05 776 782 16644747

[72] Kang S Y Jang Y Cho M S 18F-FET PET/CT as a diagnostic tool for brain abscess Clin Nucl Med 2021 46 10 e503 e506 34477604 10.1097/RLU.0000000000003741

[73] Karunanithi S Sharma P Kumar A Comparative diagnostic accuracy of contrast-enhanced MRI and (18)F-FDOPA PET-CT in recurrent glioma Eur Radiol 2013 23 09 2628 2635 23624623 10.1007/s00330-013-2838-6

[74] Fueger B J Czernin J Cloughesy T Correlation of 6-18F-fluoro-L-dopa PET uptake with proliferation and tumor grade in newly diagnosed and recurrent gliomas J Nucl Med 2010 51 10 1532 1538 20847166 10.2967/jnumed.110.078592

[75] Kracht L W Miletic H Busch S Delineation of brain tumor extent with [11C]L-methionine positron emission tomography: local comparison with stereotactic histopathology Clin Cancer Res 2004 10 21 7163 7170 15534088 10.1158/1078-0432.CCR-04-0262

[76] Tripathi M Sharma R D'Souza M Comparative evaluation of F-18 FDOPA, F-18 FDG, and F-18 FLT-PET/CT for metabolic imaging of low grade gliomas Clin Nucl Med 2009 34 12 878 883 20139821 10.1097/RLU.0b013e3181becfe0

[77] Pauleit D Floeth F Hamacher K O-(2-[18F]fluoroethyl)-L-tyrosine PET combined with MRI improves the diagnostic assessment of cerebral gliomas Brain 2005 128(Pt 3):678 687 15689365 10.1093/brain/awh399

[78] Becherer A Karanikas G Szabó M Brain tumour imaging with PET: a comparison between [18F]fluorodopa and [11C]methionine Eur J Nucl Med Mol Imaging 2003 30 11 1561 1567 14579097 10.1007/s00259-003-1259-1

[79] Chen W Silverman D HS Delaloye S 18F-FDOPA PET imaging of brain tumors: comparison study with 18F-FDG PET and evaluation of diagnostic accuracy J Nucl Med 2006 47 06 904 911 16741298

[80] Ledezma C J Chen W Sai V 18F-FDOPA PET/MRI fusion in patients with primary/recurrent gliomas: initial experience Eur J Radiol 2009 71 02 242 248 18511228 10.1016/j.ejrad.2008.04.018

[81] Pafundi D H Laack N N Youland R S Biopsy validation of 18F-DOPA PET and biodistribution in gliomas for neurosurgical planning and radiotherapy target delineation: results of a prospective pilot study Neuro-oncol 2013 15 08 1058 1067 23460322 10.1093/neuonc/not002 PMC3714146

[82] Kunz M Thon N Eigenbrod S Hot spots in dynamic (18)FET-PET delineate malignant tumor parts within suspected WHO grade II gliomas Neuro-oncol 2011 13 03 307 316 21292686 10.1093/neuonc/noq196 PMC3064604

[83] Pirotte B Goldman S Massager N Comparison of 18F-FDG and 11C-methionine for PET-guided stereotactic brain biopsy of gliomas J Nucl Med 2004 45 08 1293 1298 15299051

[84] Horsley P J Bailey D L Schembri G Hsiao E Drummond J Back M F The role of amino acid PET in radiotherapy target volume delineation for adult-type diffuse gliomas: a review of the literature Crit Rev Oncol Hematol 2025 205 104552 39521308 10.1016/j.critrevonc.2024.104552

[85] Galldiks N Stoffels G Ruge M I Role of O-(2-18F-fluoroethyl)-L-tyrosine PET as a diagnostic tool for detection of malignant progression in patients with low-grade glioma J Nucl Med 2013 54 12 2046 2054 24159047 10.2967/jnumed.113.123836

[86] Jansen N L Suchorska B Wenter V Dynamic 18F-FET PET in newly diagnosed astrocytic low-grade glioma identifies high-risk patients J Nucl Med 2014 55 02 198 203 24379223 10.2967/jnumed.113.122333

[87] Jansen N L Suchorska B Wenter V Prognostic significance of dynamic 18F-FET PET in newly diagnosed astrocytic high-grade glioma J Nucl Med 2015 56 01 9 15 25537990 10.2967/jnumed.114.144675

[88] German Glioma Network Suchorska B Jansen N L Linn J Biological tumor volume in 18FET-PET before radiochemotherapy correlates with survival in GBM Neurology 2015 84 07 710 719 25609769 10.1212/WNL.0000000000001262

[89] Galldiks N Langen K J Holy R Assessment of treatment response in patients with glioblastoma using O-(2-18F-fluoroethyl)-L-tyrosine PET in comparison to MRI J Nucl Med 2012 53 07 1048 1057 22645298 10.2967/jnumed.111.098590

[90] Galldiks N Kracht L W Burghaus L Use of 11C-methionine PET to monitor the effects of temozolomide chemotherapy in malignant gliomas Eur J Nucl Med Mol Imaging 2006 33 05 516 524 16450140 10.1007/s00259-005-0002-5

[91] Jansen N L Suchorska B Schwarz S B [18F]fluoroethyltyrosine-positron emission tomography-based therapy monitoring after stereotactic iodine-125 brachytherapy in patients with recurrent high-grade glioma Mol Imaging 2013 12 03 137 147 23490440

[92] Pöpperl G Goldbrunner R Gildehaus F J O-(2-[18F]fluoroethyl)-L-tyrosine PET for monitoring the effects of convection-enhanced delivery of paclitaxel in patients with recurrent glioblastoma Eur J Nucl Med Mol Imaging 2005 32 09 1018 1025 15877226 10.1007/s00259-005-1819-7

[93] Pöpperl G Götz C Rachinger W Serial O-(2-[(18)F]fluoroethyl)-L: -tyrosine PET for monitoring the effects of intracavitary radioimmunotherapy in patients with malignant glioma Eur J Nucl Med Mol Imaging 2006 33 07 792 800 16550381 10.1007/s00259-005-0053-7 PMC1998889

[94] Harris R J Cloughesy T F Pope W B 18F-FDOPA and 18F-FLT positron emission tomography parametric response maps predict response in recurrent malignant gliomas treated with bevacizumab Neuro-oncol 2012 14 08 1079 1089 22711609 10.1093/neuonc/nos141 PMC3408264

[95] Schwarzenberg J Czernin J Cloughesy T F Treatment response evaluation using 18F-FDOPA PET in patients with recurrent malignant glioma on bevacizumab therapy Clin Cancer Res 2014 20 13 3550 3559 24687922 10.1158/1078-0432.CCR-13-1440 PMC4079729

[96] Galldiks N Rapp M Stoffels G Response assessment of bevacizumab in patients with recurrent malignant glioma using [18F]Fluoroethyl-L-tyrosine PET in comparison to MRI Eur J Nucl Med Mol Imaging 2013 40 01 22 33 23053325 10.1007/s00259-012-2251-4

[97] Hutterer M Nowosielski M Putzer D O-(2-18F-fluoroethyl)-L-tyrosine PET predicts failure of antiangiogenic treatment in patients with recurrent high-grade glioma J Nucl Med 2011 52 06 856 864 21622893 10.2967/jnumed.110.086645

[98] Herrmann K Czernin J Cloughesy T Comparison of visual and semiquantitative analysis of 18F-FDOPA-PET/CT for recurrence detection in glioblastoma patients Neuro-oncol 2014 16 04 603 609 24305722 10.1093/neuonc/not166 PMC3956344

[99] Walter F Cloughesy T Walter M A Impact of 3,4-dihydroxy-6-18F-fluoro-L-phenylalanine PET/CT on managing patients with brain tumors: the referring physician's perspective J Nucl Med 2012 53 03 393 398 22323780 10.2967/jnumed.111.095711

[100] Kebir S Fimmers R Galldiks N Late pseudoprogression in glioblastoma: diagnostic value of dynamic O-(2-[18F]fluoroethyl)-L-tyrosine PET Clin Cancer Res 2016 22 09 2190 2196 26673798 10.1158/1078-0432.CCR-15-1334

[101] Palmisciano P Watanabe G Conching A The role of [ ^68^ Ga]Ga-DOTA-SSTR PET radiotracers in brain tumors: a systematic review of the literature and ongoing clinical trials Cancers (Basel) 2022 14 12 2925 35740591 10.3390/cancers14122925 PMC9221214

[102] Valotassiou V Leondi A Angelidis G Psimadas D Georgoulias P SPECT and PET imaging of meningiomas ScientificWorldJournal 2012 2012 412580 22623896 10.1100/2012/412580 PMC3353476

[103] Graf R Plotkin M Steffen I G Magnetic resonance imaging, computed tomography, and 68Ga-DOTATOC positron emission tomography for imaging skull base meningiomas with infracranial extension treated with stereotactic radiotherapy—a case series Head Face Med 2012 8 1 22217329 10.1186/1746-160X-8-1 PMC3274469

[104] Law W P Fiumara F Fong W Macfarlane D J The “double pituitary hot spot” sign of skull base meningioma on gallium-68-labelled somatostatin analogue PET J Med Imaging Radiat Oncol 2013 57 06 680 683 24283556 10.1111/1754-9485.12069

[105] Purandare N C Puranik A Shah S Differentiating dural metastases from meningioma: role of 68Ga DOTA-NOC PET/CT Nucl Med Commun 2020 41 04 356 362 31939900 10.1097/MNM.0000000000001155

[106] Unterrainer M Ruf V Ilhan H 68Ga-DOTATOC PET/CT differentiates meningioma from dural metastases Clin Nucl Med 2019 44 05 412 413 30829858 10.1097/RLU.0000000000002513

[107] Gehler B Paulsen F Oksüz M O [68Ga]-DOTATOC-PET/CT for meningioma IMRT treatment planning Radiat Oncol 2009 4 56 19922642 10.1186/1748-717X-4-56 PMC2785827

[108] Nyuyki F Plotkin M Graf R Potential impact of (68)Ga-DOTATOC PET/CT on stereotactic radiotherapy planning of meningiomas Eur J Nucl Med Mol Imaging 2010 37 02 310 318 19763565 10.1007/s00259-009-1270-2

[109] d'Amico A Stąpór-Fudzińska M Tarnawski R CyberKnife radiosurgery planning of a secreting pituitary adenoma performed with ^68^ Ga DOTATATE PET and MRI Clin Nucl Med 2014 39 12 1043 1044 25140540 10.1097/RLU.0000000000000535

[110] Zhao X Xiao J Xing B Wang R Zhu Z Li F Comparison of (68)Ga DOTATATE to 18F-FDG uptake is useful in the differentiation of residual or recurrent pituitary adenoma from the remaining pituitary tissue after transsphenoidal adenomectomy Clin Nucl Med 2014 39 07 605 608 24873787 10.1097/RLU.0000000000000457

[111] Xiao J Zhu Z Zhong D Ma W Wang R Improvement in diagnosis of metastatic pituitary carcinoma by 68Ga DOTATATE PET/CT Clin Nucl Med 2015 40 02 e129 e131 24873798 10.1097/RLU.0000000000000462

[112] Kaya G Soydas Turan B Dagdelen S Berker M Tuncel M 68Ga-DOTATATE PET/CT in pituitary carcinoma Clin Nucl Med 2021 46 12 996 998 34269734 10.1097/RLU.0000000000003804

[113] van den Bent M J Vogelbaum M A Cloughesy T Response assessment in neuro-oncology (RANO) 2009-2025: broad scope and implementation—a progress report Neuro-oncol 2025 27 09 2209 2224 40317212 10.1093/neuonc/noaf118 PMC12526114

[114] Wen P Y Macdonald D R Reardon D A Updated response assessment criteria for high-grade gliomas: response assessment in neuro-oncology working group J Clin Oncol 2010 28 11 1963 1972 20231676 10.1200/JCO.2009.26.3541

[115] Macdonald D R Cascino T L Schold S C Jr Cairncross J G Response criteria for phase II studies of supratentorial malignant glioma J Clin Oncol 1990 8 07 1277 1280 2358840 10.1200/JCO.1990.8.7.1277

[116] Response Assessment in Neuro-Oncology (RANO) group Lin N U Lee E Q Aoyama H Response assessment criteria for brain metastases: proposal from the RANO group Lancet Oncol 2015 16 06 e270 e278 26065612 10.1016/S1470-2045(15)70057-4

[117] Chamberlain M Junck L Brandsma D Leptomeningeal metastases: a RANO proposal for response criteria Neuro-oncol 2017 19 04 484 492 28039364 10.1093/neuonc/now183 PMC5464328

[118] Okada H Weller M Huang R Immunotherapy response assessment in neuro-oncology: a report of the RANO working group Lancet Oncol 2015 16 15 e534 e542 26545842 10.1016/S1470-2045(15)00088-1 PMC4638131

[119] Kim S H Roytman M Madera G Evaluating diagnostic accuracy and determining optimal diagnostic thresholds of different approaches to [ ^68^ Ga]-DOTATATE PET/MRI analysis in patients with meningioma Sci Rep 2022 12 01 9256 35661809 10.1038/s41598-022-13467-9 PMC9166786

[120] Wen P Y van den Bent M Youssef G RANO 2.0: update to the response assessment in neuro-oncology criteria for high- and low-grade gliomas in adults J Clin Oncol 2023 41 33 5187 5199 37774317 10.1200/JCO.23.01059 PMC10860967

[121] Lohmann P Galldiks N Kocher M Radiomics in neuro-oncology: basics, workflow, and applications Methods 2021 188 112 121 32522530 10.1016/j.ymeth.2020.06.003

[122] Kickingereder P Bonekamp D Nowosielski M Radiogenomics of glioblastoma: machine learning-based classification of molecular characteristics by using multiparametric and multiregional MR imaging features Radiology 2016 281 03 907 918 27636026 10.1148/radiol.2016161382

[123] Lohmann P Lerche C Bauer E K Predicting IDH genotype in gliomas using FET PET radiomics Sci Rep 2018 8 01 13328 30190592 10.1038/s41598-018-31806-7 PMC6127131

[124] Franco P Würtemberger U Dacca K SPectroscOpic prediction of bRain Tumours (SPORT): study protocol of a prospective imaging trial BMC Med Imaging 2020 20 01 123 33228567 10.1186/s12880-020-00522-y PMC7685595

[125] Zhou H Chang K Bai H X Machine learning reveals multimodal MRI patterns predictive of isocitrate dehydrogenase and 1p/19q status in diffuse low- and high-grade gliomas J Neurooncol 2019 142 02 299 307 30661193 10.1007/s11060-019-03096-0 PMC6510979

[126] Peng L Parekh V Huang P Distinguishing true progression from radionecrosis after stereotactic radiation therapy for brain metastases with machine learning and radiomics Int J Radiat Oncol Biol Phys 2018 102 04 1236 1243 30353872 10.1016/j.ijrobp.2018.05.041 PMC6746307

[127] Kocak B Mese I Ates Kus E Radiomics for differentiating radiation-induced brain injury from recurrence in gliomas: systematic review, meta-analysis, and methodological quality evaluation using METRICS and RQS Eur Radiol 2025 35 08 4490 4505 39937273 10.1007/s00330-025-11401-x PMC12226684

[128] Hollon T C Pandian B Adapa A R Near real-time intraoperative brain tumor diagnosis using stimulated Raman histology and deep neural networks Nat Med 2020 26 01 52 58 31907460 10.1038/s41591-019-0715-9 PMC6960329

[129] Lohmann P Franceschi E Vollmuth P Radiomics in neuro-oncological clinical trials Lancet Digit Health 2022 4 11 e841 e849 36182633 10.1016/S2589-7500(22)00144-3

[130] Galldiks N Langen K J Albert N L Investigational PET tracers in neuro-oncology—What's on the horizon? A report of the PET/RANO group Neuro-oncol 2022 24 11 1815 1826 35674736 10.1093/neuonc/noac131 PMC9629449

[131] Chung B T Chen H Y Gordon J First hyperpolarized [2- ^13^ C]pyruvate MR studies of human brain metabolism J Magn Reson 2019 309 106617 31648132 10.1016/j.jmr.2019.106617 PMC6880930

[132] Izquierdo-Garcia J L Cai L M Chaumeil M M Glioma cells with the IDH1 mutation modulate metabolic fractional flux through pyruvate carboxylase PLoS One 2014 9 09 e108289 25243911 10.1371/journal.pone.0108289 PMC4171511

[133] Kim Y Chen H Y Nickles T Translation of hyperpolarized [ ^13^ C, ^15^ N _2_ ]urea MRI for novel human brain perfusion studies Npj Imaging 2025 3 01 11 40124419 10.1038/s44303-025-00073-3 PMC11925798

[134] Eskandari R Kim N Mamakhanyan A Hyperpolarized [5- ^13^ C,4,4- ^2^ H _2_ ,5- ^15^ N]-L-glutamine provides a means of annotating in vivo metabolic utilization of glutamine Proc Natl Acad Sci U S A 2022 119 19 e2120595119 35512101 10.1073/pnas.2120595119 PMC9172133

[135] Patel S Porcari P Coffee E Simultaneous noninvasive quantification of redox and downstream glycolytic fluxes reveals compartmentalized brain metabolism Sci Adv 2024 10 51 eadr2058 39705365 10.1126/sciadv.adr2058 PMC11661454

[136] Michaelson N M Watsula A Bakare-Okpala A Mohamadpour M Chukwueke U N Budhu J A Disparities in neuro-oncology Curr Neurol Neurosci Rep 2023 23 12 815 825 37889427 10.1007/s11910-023-01314-x

[137] Mukherjee D Zaidi H A Kosztowski T Disparities in access to neuro-oncologic care in the United States Arch Surg 2010 145 03 247 253 20231625 10.1001/archsurg.2009.288

[138] Kim Y Armstrong T S Gilbert M R Celiku O Disparities in the availability of and access to neuro-oncology trial-supporting infrastructure in the United States J Natl Cancer Inst 2025 117 03 511 516 39325856 10.1093/jnci/djae240 PMC11884859

[139] Budhu J A Chukwueke U N Jackson S Defining interventions and metrics to improve diversity in CNS clinical trial participation: A SNO and RANO effort Neuro-oncol 2024 26 04 596 608 38071654 10.1093/neuonc/noad242 PMC10995510

[140] Wen P Y van den Bent M Youssef G RANO 2.0: update to the response assessment in neuro-oncology criteria for high- and low-grade gliomas in adultsJ Clin Oncol2023 41(33). Accessed August 1, 2025 at:https://ascopubs.org/doi/10.1200/JCO.23.01059 10.1200/JCO.23.01059 PMC10860967 37774317

